# Erratum: Origin of multiple band gap values in single width nanoribbons

**DOI:** 10.1038/srep38119

**Published:** 2016-11-30

**Authors:** Deepika, Shailesh Kumar, Alok Shukla, Rakesh Kumar

Scientific Reports 6: Article number: 3616810.1038/srep36168; published online: 11 03 2016; updated: 11 30 2016

The original version of this Article contained errors in the name of the author Deepika, which was incorrectly given as Deepika Goyal.

These errors have now been corrected in the PDF and HTML versions of the Article.

