# Supplementary material for: Origin of multiple band gap values in single width nanoribbons
Source: Sci Rep. 2016 Nov 3;6:36168. doi: 10.1038/srep36168 (PMC5093554; doi:10.1038/srep36168)
Supplement: Supplementary Information [file srep36168-s1.pdf]

Supplementary for "Origin of multiple band gap values in single width nanoribbons"

Deepika[1], Shailesh Kumar[2], Alok Shukla[3], and Rakesh Kumar[1,*]

[1]Department of Physics, Indian Institute of Technology Ropar, Rupnagar-140001, India
[2]School of Chemistry, Physics and Mechanical Engineering, Queensland University of Technology, Brisbane, Queensland 4000, Australia
[3]Department of Physics, Indian Institute of Technology Bombay, Powai, Mumbai-400076, India
[*]corresponding.rakesh@iitrpr.ac.in

ABSTRACT

Deterministic band gap in quasi-one-dimensional nanoribbons is prerequisite for their integrated functionalities in high-performance molecular-electronics based devices. However, multiple band gap values commonly observed in the same width of graphene nanoribbons fabricated in same slot of the experiments remains unresolved, and raise a critical concern over scalable production of pristine and/or hetero-structure nanoribbons with deterministic properties and functionalities for plethora of applications. Here, we show that a modification in the depth of potential wells in the periodic direction of a supercell on relative shifting of passivating atoms at the edges is the origin of multiple band gap values for the same width of nanoribbons in a crystallographic orientation, although they carry practically the same ground state energy. The results are similar when calculations are extended from planar graphene to buckled silicene nanoribbons. Thus, the findings facilitate tuning of the electronic properties of quasi-one-dimensional materials such as bio-molecular chains, organic and inorganic nanoribbons by performing edge engineering.

S1. Energetically favorable edge configurations

To justify the considered edge configurations of GNRs supercell to be energetically favorable, we compared their ground state energy with supercell of another possible edge configuration having the same number of atoms of each type. For ZGNRs, our proposed edge configurations config. I and config. II are shown in Fig. 1 (a) and (b) respectively, while another possible edge configuration having the same number of atoms of each type is shown in Fig. 1 (c). We observed that our proposed edge configurations are energetically favorable than another possible edge configuration. The ground state energy for typical supercell of 5-ZGNRs of our edge configuration is E (config. I) = -272.68(5) eV and E (config. II) = -272.66(7) eV while for the supercell corresponding to Fig. 1 (c) is E = - 264.47(0) eV. It clearly shows that our proposed edge configuration for ZGNRs is energetically favorable.

Similarly for AGNRs, we compared our edge configuration (config. I and config. II) shown in Fig. 2 (a), (b) with another edge configuration shown in Fig. 2 (c). We observe that our configurations are energetically favorable than another possible edge configuration. The ground state energy for typical supercell of 5-AGNRs corresponding to our proposed edge configurations is E (config. I) = - 197.04(0) eV and E (config. II) = - 196.38(8) eV, while for the configuration corresponding to Fig. 2 (c) is E = -187.74(2) eV. It clearly shows that our proposed edge configuration for AGNRs is energetically favorable.

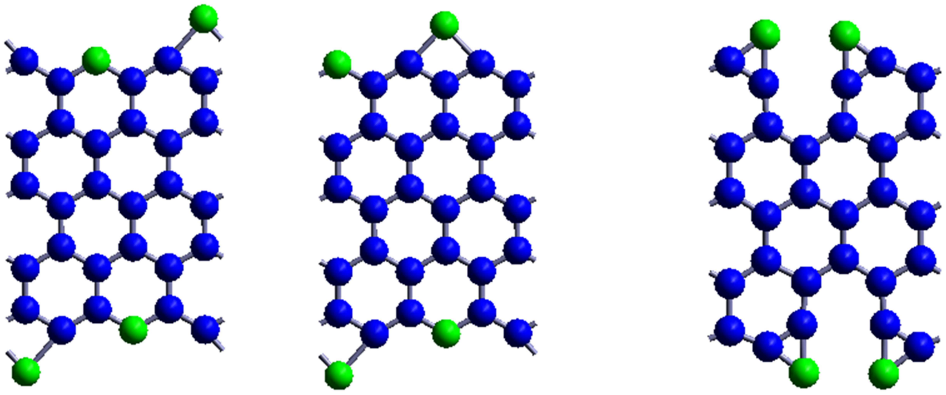

Config. I
E = -272.68(5) eV

Config. II
E = -272.66(7) eV

Other possible
edge configuration
E = -264.47(0) eV

(a) (b) (c)

Figure 1. Supercell edge configurations of oxygen passivated 5-ZGNRs (a) Config. I (b) Config. II proposed in the manuscript, (c) another equivalent possible edge configuration having the same number of atoms of each type. Blue and green spheres represent carbon and oxygen atoms, respectively.

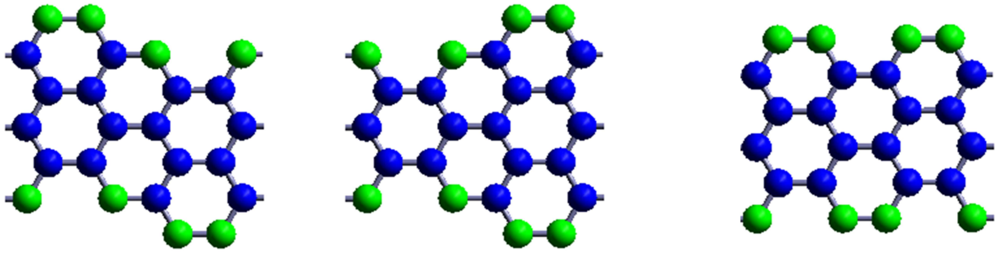

Config. I
E = -197.04(0)eV

Config. II
E = -196.38(8) eV

Other possible
edge configuration
E = -187.74(2) eV

(a) (b) (c)

Figure 2. Supercell edge configurations of oxygen passivated 5-AGNRs (a) Config. I (b) Config. II proposed in the manuscript, (c) another equivalent possible edge configuration having the same number of atoms of each type. Blue and green spheres represent carbon and oxygen atoms, respectively.

S2. Band structure of Zigzag GNRs

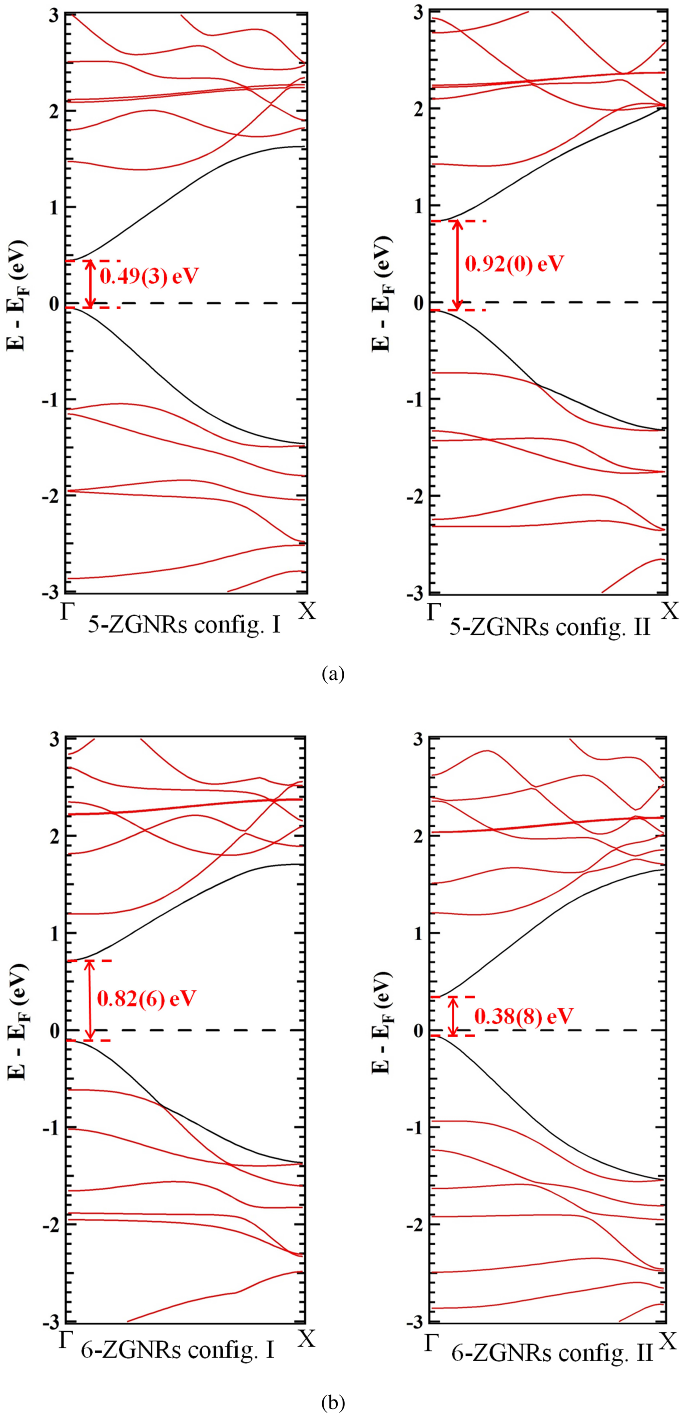

Figure 3. Band structure plots for config. I and config. II of (a) 5-ZGNRs and (b) 6-ZGNRs from Γ-point (k = 0) to X-point (k = π). $E - E_F$ is the energy with reference to the Fermi level. Note: a change in the magnitude of the band gap values in config. I and config. II.

S3. Band structure of Armchair GNRs

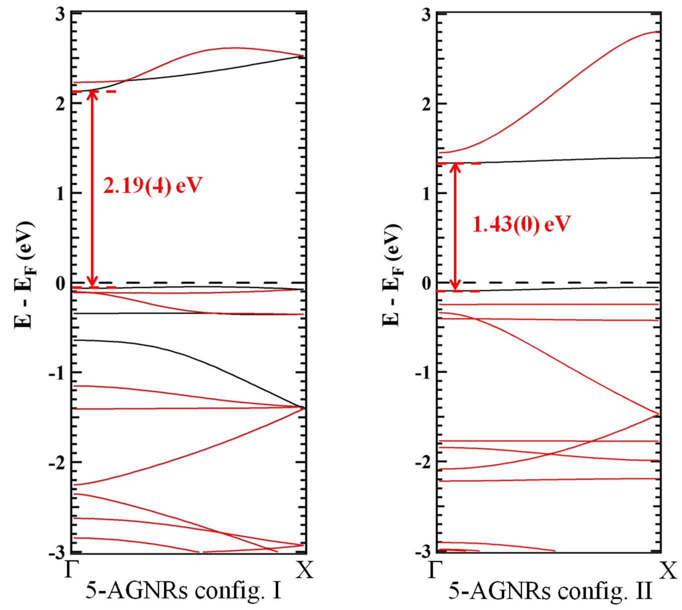

(a)

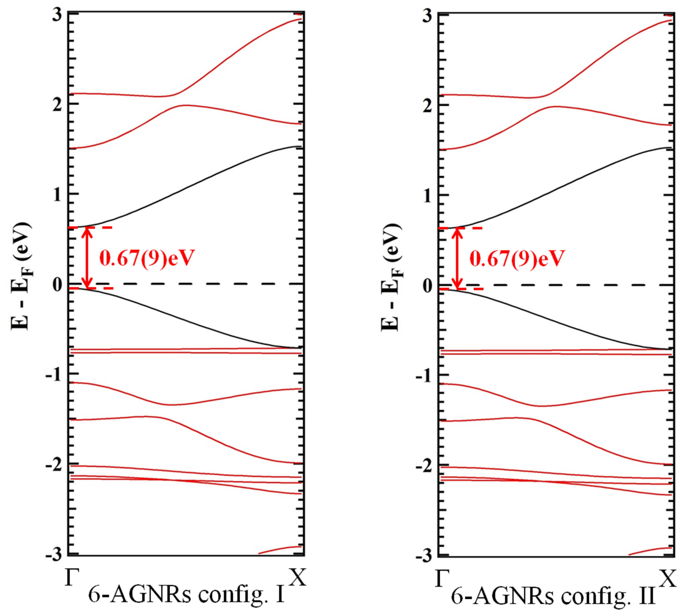

(b)

Figure 4. Band structure plots for config. I and config. II of (a) 5-AGNRs and (b) 6-AGNRs from Γ-point (k = 0) to X-point (k = π). $E - E_F$ is the energy with reference to the Fermi level. Note: a change in the magnitude of the band gap values in config. I and config. II.

S4. Investigation of the exceptional behavior for N_a = 5 and 7-AGNRs

To investigate the exceptional behavior for N_a = 5 and 7, inter-atomic distances are examined near the edges of AGNRs supercells. It is observed that the respective inter-atomic distance at the edges for both the configurations are nearly same in AGNRs except for N_a = 5 and 7. The inter-atomic distance at the edges for N_a = 5 and 7 are found to be contracted at one position and expanded at another position along the periodic direction. The contraction and expansion of the distance modifies the electrostatic interactions between atoms at the edges as well as intra-edge positions, which is reflected from unexpected relative increase in the difference of the ground state energies for N_a = 5 and 7 with respect to other AGNRs (N_a > 7). The exceptional change in difference of the ground state energy for N_a = 5 and 7 results from modifications in the intra and inter edge electrostatic interactions in sub-nanometer ribbons. Therefore, the depth of the deepest potential well for the configurations cannot be correlated with band gap values for N_a = 5 and 7.

S5: Silicene nanoribbon passivated with oxygen atoms

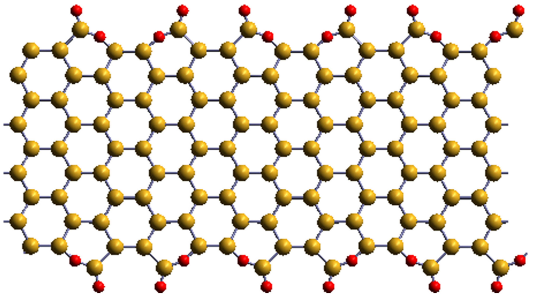

(a) Top View

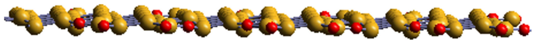

(b) Side View

9-ASiNRs config. I

Figure 5. Buckled silicene nanoribbons in armchair orientations (a) top view and (b) side view of 9-ASiNRs config. I. Gold and red spheres represent silicene and oxygen atoms, respectively.

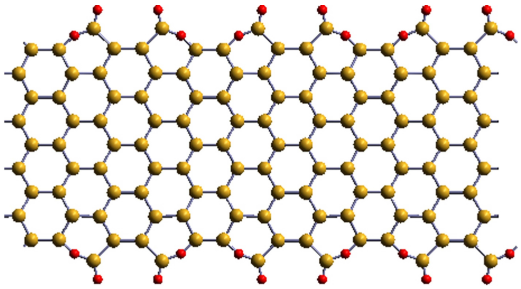

(a) Top View

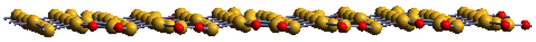

(b) Side View

9-ASiNRs config. II

Figure 6. Buckled silicene nanoribbons in armchair orientations (a) top view and (b) side view of 9-ASiNRs config. II. Gold and red spheres represent silicene and oxygen atoms, respectively.

S6. Graphene Nanoribbons passivated at the edges with hydrogen and oxygen atoms

In order to verify the concept of multiple band gaps for nanoribbons passivated with different types of atoms at the edges, we consider GNRs passivated with two different types of atoms such as hydrogen and oxygen. Typical two edge configurations (config. I and config. II) for 7-AGNRs are shown in Fig. 7. Similar to the GNRs passivated with oxygen atoms, multiple band gap values are observed for the same width of GNRs passivated with hydrogen and oxygen having practically the same ground state energy. The band gap values and ground state energy for both the configuration of 7-AGNRs passivated at multiple functional groups at the edges are tabulated in Table 1

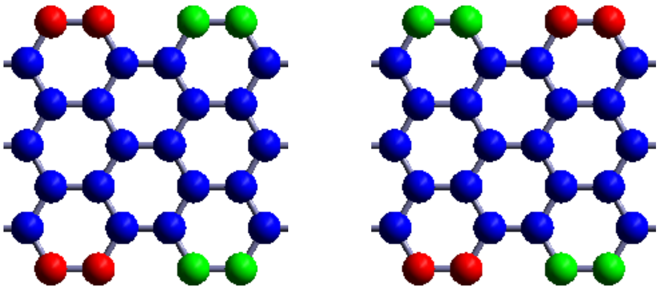

(a) (b)

Figure 7. Two edge configurations for a typical nanoribbons (N_a = 7) passivated with the same number of hydrogen and oxygen atoms at the edges, (a) config. I and (b) config. II. Blue, green and red spheres represent carbon, hydrogen and oxygen atoms respectively.

Table 1. Ground state energy and band gap values for the config. I and config. II of hydrogen and oxygen passivated 7-AGNRs as shown in Fig. 8.

	config. I	config. II
Total no. of atoms, (C,H,O)	(20,4,4)	(20,4,4)
Ground state energy (eV)	-236.49(3)	-236.58(8)
Band gap (eV)	0.50(7)	0.63(2)

S7. Rough edged graphene nanoribbons

Rough edged GNRs are designed by combination of zigzag and armchair edges. Relative shift of atoms at the edges is not possible as considered earlier for ZGNRs and AGNRs, therefore two edge configurations are created with interchange of atoms at the edges. The ground state energy of both the configurations is practically same, while a significant difference in the band gap values is observed [Table. 2]. Therefore, the probability for the formation of both configurations is nearly same with multiple band gap values in the experiments.

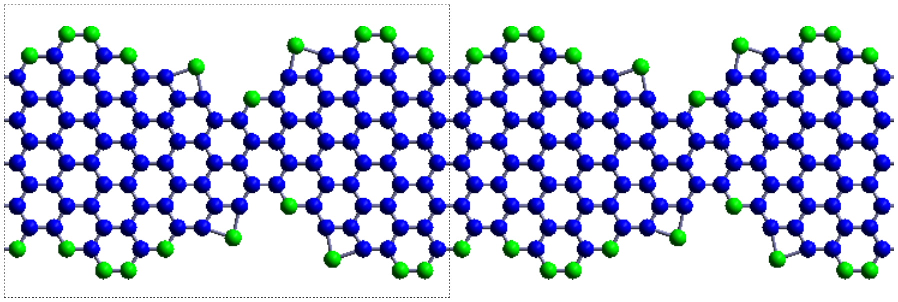

(a)

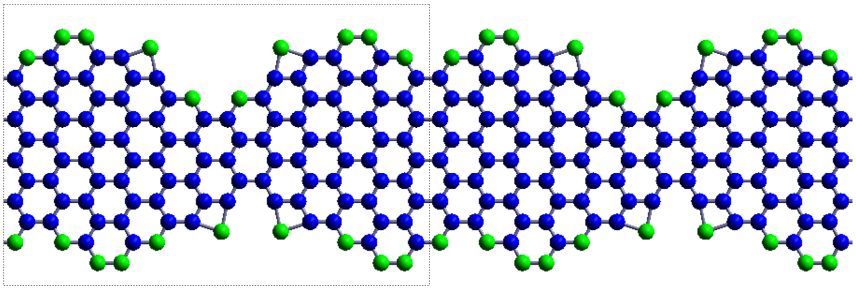

(b)

Figure 8. Two edge configurations (a) config. I and (b) config. II for oxygen passivated rough edged GNRs with the same no. of atoms of each type in the supercells. Blue and green spheres represent carbon and oxygen atoms, respectively. Dotted rectangular box represents the supercells for rough edged GNRs

Table 2. Ground state energy and band gap values for rough edged GNRs as shown in Fig. 9.

	Config. I	Config. II
Total no. of atoms, (C, O)	(100, 20)	(100, 20)
Ground state energy (eV)	-1026.53(7)	-1026.57(7)
Band gap (eV)	0.35(4)	0.42(6)
